# Light-Driven CO_2_ Reduction by Co-Cytochrome *b*_562_

**DOI:** 10.3389/fmolb.2021.609654

**Published:** 2021-04-15

**Authors:** Rafael Alcala-Torano, Nicholas Halloran, Noah Gwerder, Dayn J. Sommer, Giovanna Ghirlanda

**Affiliations:** School of Molecular Sciences, Arizona State University, Tempe, AZ, United States

**Keywords:** cobalt porphyrin, Carbon fixation, CO_2_ reduction, catalysis, protein design, proton reduction

## Abstract

The current trend in atmospheric carbon dioxide concentrations is causing increasing concerns for its environmental impacts, and spurring the developments of sustainable methods to reduce CO_2_ to usable molecules. We report the light-driven CO_2_ reduction in water in mild conditions by artificial protein catalysts based on cytochrome *b*_562_ and incorporating cobalt protoporphyrin IX as cofactor. Incorporation into the protein scaffolds enhances the intrinsic reactivity of the cobalt porphyrin toward proton reduction and CO generation. Mutations around the binding site modulate the activity of the enzyme, pointing to the possibility of further improving catalytic activity through rational design or directed evolution.

## Introduction

The ongoing use of fossil fuels has led to an increase in atmospheric CO_2_ concentrations, causing severe consequences for the environment (Lacis et al., 2010). Current research efforts are focused on developing energetic alternatives that can help curb CO_2_ emissions. Although nature aids in the removal of this greenhouse gas through photosynthesis (Sun et al., 2014; Kondo et al., 2018), negative emissions technologies are necessary to reduce the excess gas from the atmosphere (Smith et al., 2015). One possible path is through artificial photosynthesis, where light is utilized as the energy source to drive production of carbon-free or carbon-neutral fuels, mirroring the role of the enzyme RuBisCO in fixing CO_2_. In that vein, promising approaches interfacing photosensitizers and/or materials with molecular catalysts, enzymes, and microorganisms have been developed (Woo, 2017; Luan and Lu, 2018; Edwards and Bren, 2020; Ma et al., 2020).

Artificial metalloenzymes obtained by incorporating organometallic catalysts within a protein bridge traditional molecular catalysts and enzymes, with the advantage of expanding the range of reactivity encountered in natural enzymes. Protein scaffolds may aide the efficiency of the organometallic center by offering tunable primary and secondary coordination spheres, by facilitating reactant binding and product release to and from the active site, and by protecting the organometallic center from degradation (Lu et al., 2009; Alcala-Torano et al., 2016; Schwizer et al., 2018; Davis and Ward, 2019; Nastri et al., 2019; Oohora et al., 2019; Vornholt et al., 2020). This approach has been used to produce water-soluble catalysts that are capable of producing hydrogen from protons under mild conditions, repurposing a diverse group of organometallic catalysts (Sano et al., 2011; Roy et al., 2012; Kleingardner et al., 2014; Onoda et al., 2014; Sommer et al., 2014; Sommer et al., 2016; Firpo et al., 2018; Call et al., 2019a; Slater et al., 2019; Walsh et al., 2019; Alvarez-Hernandez et al., 2020; Laureanti et al., 2020; Le et al., 2020; Roy et al., 2020). Comparatively little work has been carried out on the reduction of CO_2_ by hybrid metalloenzymes: in one example nickel cyclam complexes anchored to azurin support catalytic CO_2_ reduction, with evidence of protein modulation of activity compared to the isolated cyclam (Schneider and Shafaat, 2016; Liu et al., 2018; Schneider et al., 2018).

Here, we investigate whether coordination of CoPPIX within a protein scaffold would support carbon dioxide reduction, motivated by recent reports of metalloporphyrins as molecular CO_2_ reduction electrocatalysts (Manbeck and Fujita, 2015; Mondal et al., 2015; Zhang et al., 2017; Birdja et al., 2018; Rao et al., 2018) and most recently photocatalysts (Call et al., 2019b; Fukuzumi et al., 2019). In these catalysts, tuning the reaction conditions or modifying the porphyrin framework in order to stabilize catalytic intermediates through hydrogen bonding resulted in increased activities of these catalysts toward CO_2_ reduction (Bhugun et al., 1996; Lee et al., 2011; Costentin et al., 2012; Azcarate et al., 2016a; b; Call et al., 2019b). Further, increased turnover numbers (TON) and product selectivity upon incorporation of cobalt porphyrins into supramolecular structures such as metal-organic frameworks (Kornienko et al., 2015; Lin et al., 2015; Zhang et al., 2016; Feng et al., 2020; Wu et al., 2020; Zhang et al., 2020) and polymers (Kramer and McCrory, 2016; Liu and McCrory, 2019) suggest that catalysis by cobalt porphyrins could be enhanced by incorporation into a protein environment. Our approach utilizes a natural protein scaffold, cytochrome *b*_562_ (cyt *b*_562_), in which the native heme has been swapped with its cobalt analog, cobalt protoporphyrin IX (CoPPIX). Our group and others have previously demonstrated that exchanging the metal ion to cobalt in heme-binding proteins or peptides results in metalloproteins that can catalyze proton reduction activity, and show an increase of H_2_ production compared to the cobalt porphyrin in solution. Moreover, altering the protein sequence results in fine modulation of the total activity, further supporting the crucial role of the protein environment throughout the catalytic cycle, beyond simple encapsulation (Kleingardner et al., 2014; Sommer et al., 2014; Sommer et al., 2016; Firpo et al., 2018; Le and Bren, 2019; Alvarez-Hernandez et al., 2020; Le et al., 2020). Compared with other supramolecular systems, proteins are easily modifiable by mutating the amino acid sequence, allowing the systematic exploration of the primary and secondary coordination sphere. Recent advances dramatically expand the range of chemical moieties available on the side chains through incorporation of noncanonical amino acids (Alcala-Torano et al., 2016; Burke et al., 2019; Drienovská et al., 2020; Drienovská and Roelfes, 2020; Zhou and Roelfes, 2020). Further, proteins can be optimized by directed evolution coupled with high-thrughput screening to identify favorable mutations (Liang et al., 2019; Chen and Arnold, 2020; Jeong et al., 2020; Markel et al., 2020).

## Material and Methods

All chemicals were purchased from Sigma-Aldrich and used without further purification unless otherwise noted. Calibration gases were obtained from Matheson in 14 L lecture bottles. All aqueous solutions were prepared using deionized water with a resistivity greater or equal to 18 MΩ. Cobalt (III) protoporphyrin IX chloride was purchased from Sigma-Aldrich and used without further purification.

### Protein Expression

Mutants were generated in a pET30c (+) vector encoding WT cyt *b*_562_ using mutagenic primers by Gibson assembly as described in Supplementary Information (Supplementary Table S1 and Supplementary Figure S8) (Sommer et al., 2016). The verified mutants were transformed into *Escherichia coli* BL21 (DE3) and grown in 1 L of 2xTY media at 37 °C with shaking at 300 rpm. Cells were induced with 1 mM IPTG at an OD_600_ of 0.6 and harvested after 4 h of expression. The cell pellets were suspended in 20 mM Tris-HCl, 1 mM DTT, 0.5 mM EDTA and lysed by multiple cycles of ultrasonication. The clarified lysate was brought to 75% saturation with solid ammonium sulfate, and precipitated proteins were removed by centrifugation. The supernatant, containing the cytochrome mutants, was dialyzed against two changes of 10 mM Tris pH 7.5 and one of water at 4 °C. Following dialysis, the protein solution was lyophilized and redissolved in 10 mM NaCl water for further purification via RP-HPLC, using a semi-preparatory scale C18 column with a linear 1% min^−1^ gradient from 100% solvent A (0.1% v/v TFA in water) to 100% solvent B (4.9% v/v water, 0.1% v/v TFA in acetonitrile). The fractions containing the desired protein were then lyophilized to yield the pure apo-protein. The protein identities were confirmed via MALDI-TOF-MS and their purity determined by C18 analytical analysis (Supplementary Information, Supplementary Figure S9). The purified protein was lyophilized and stored at −78 °C. Protein stocks were prepared by dissolving the protein in the appropriate phosphate buffer. The protein stock concentration was determined by UV spectroscopy in 6 M urea using an extinction coefficient of 2980 M^−1^ cm^−1^ at 280 nm.

### Binding Assays

The dissociation constant (*K*_d_) of each mutant was assessed by titrating a solution of CoPPIX in 200 mM potassium phosphate buffer at pH 7.5 with a solution of the apo protein, and monitoring the absorbance of the Soret peak corresponding to the holo protein (∼425 nm depending on the mutant) (Supplementary Figure S2). The initial CoPPIX concentration was determined using the observed absorbance value at the Soret peak of the free porphyrin (417 nm) and ε = 143,540 M^−1^ cm^−1^ (as determined by ICP-OES experiments). All titrations were carried out in an anaerobic chamber (Coy Laboratory Products) equipped with UV-Vis (Ocean Optics USB4000). Data were fitted to Eq. 1 using OriginLab as described in the Supplementary Information by adapting established procedures (Hulme and Trevethick, 2010; Kovacs et al., 2010; Thordarson, 2011).

$$\Delta A=\frac{\Delta \varepsilon }{2}\left({\left[Cyt\right]}_{T}+{\left[CoPPIX\right]}_{T}+K_{d}-\sqrt{{\left({\left[Cyt\right]}_{T}+{\left[CoPPIX\right]}_{T}+K_{d}\right)}^{2}-4{\left[CoPPIX\right]}_{T}{\left[Cyt\right]}_{T}}\right),$$ (1)

Δ*A* and Δε are the differences in absorbance and in extinction coefficients, respectively, between the free porphyrin and the holo protein at the latter’s Soret peak (∼425 nm). [CoPPIX]_T_ is the total CoPPIX concentration, and [Cyt]_T_ is the total protein concentration after each addition.

### Circular Dichroism Spectroscopy

CD spectra were recorded on a JASCO J-815 spectropolarimeter in the range of 200–280 nm using a 1 mm cuvette. Data points were recorded every 1 nm and averaged over three scans. The protein concentration was kept at 5 µM in 10 mM phosphate buffer pH 7.5 and the spectra was recorded at 20 °C. Holo protein measurements were carried out in excess CoPPIX (20 μM). Thermal denaturation was performed by heating samples from 4 to 90 °C at a rate of 1°C min^−1^, monitoring the loss of signal at 222 nm.

### Photocatalytic Experiments

Reactions were carried out in sealed cuvettes. Stock buffer containing 200 mM potassium phosphate and 125 mM ascorbic acid at pH 6.0 was bubbled with either CO_2_ or Ar, and adjusted back to pH 6.0 with aqueous KOH if necessary. Finally, solid [Ru (bpy)_3_]Cl_2_·6 H_2_O was added to a 1.25 mM final concentration, flash frozen in aliquots, and stored at −80 °C. Before each assay, CoPPIX was dissolved in 100 mM KOH to make a saturated solution, and the stock concentration was determined by UV-Vis using the Soret peak at 417 nm with an extinction coefficient of ε = 143,540 M^−1^ cm^−1^ (as determined by ICP-OES measurements). The frozen buffer was thawed under an atmosphere of either CO_2_ or Ar, and CoPPIX and/or protein (in 200 mM potassium phosphate) added to obtain working solutions containing 20 µM CoPPIX, 30 µM protein, 100 mM ascorbic acid, and 1 mM [Ru(bpy)_3_]^2+^ in 200 mM potassium phosphate. The ratio of CoPPIX to apo protein was chosen from equilibrium calculations based on the highest observed *K*_d_ value to ensure ≥95% of porphyrin was bound to the protein.

For each experiment, 400 µL of sample was added to a 10 mm × 1 mm gas tight cuvette of known headspace volume, and the headspace sparged with gas (Ar or CO_2_) for 20 min. The cuvettes were then irradiated with a white light LED source for 8 h. All experiments were done in triplicate and the variation is reported as the standard deviation of the sample. The gaseous products, H_2_ and CO, were quantified by removing 100 μL from the headspace at time intervals, and analyzed with an SRI Instruments gas chromatograph equipped with a 3 ft × 1/8” molecular sieve 5 Å packed column. H_2_ and CO were detected and quantified simultaneously from the same injection using a Thermal Conductivity Detector (TCD) and a Flame ionization Detector (FID) with a methanizer connected in series. The analytes were eluted using Ar as a carrier gas with a temperature program starting at 60 °C for 1 min, ramping at 20 °C min^−1^ until 80°C, holding for 2 min, ramping at 50 °C min^−1^ to 250°C, and holding until the CO_2_ exited the instrument, with a retention time (*t*_R_) ca. 12 min. A peak corresponding to H_2_ was seen on the TCD channel at *t*_R_ of 0.400 min, while the peak corresponding to CO appeared at *t*_R_ = 3.42 min on the FID channel. Peak areas were converted to moles using a calibration curve (Supplementary Figures S4 and S5). After irradiation was stopped, the solution was frozen at −80 °C for future analysis. Formate was quantified by diluting the sample to 10% v/v D_2_O in water and 100 µM sodium 4,4-dimethyl-1-silapentane-1- sulfonate (DSS) as an internal standard. The samples were then analyzed by ^1^H NMR using a water suppression method with 64 scans and a 30 s relaxation delay. The formate concentration was determined by comparing the integration area of the singlet at 8.45 ppm to the DSS peak at 0.0 ppm (Boston et al., 2013; Yehezkeli et al., 2016).

Turnover numbers (TON) are reported as the ratio of moles of product produced by moles of total CoPPIX present in solution. Comparisons between the means of each experiment were assessed using a two-tailed *t*-test analysis for samples with equal or unequal variances as determined by Levene’s test. The null hypothesis (H_0_: ${\bar{x}}_{1}-{\bar{x}}_{2}=0$) was rejected for *p*-values < 0.05. The calculated *p*-values can be found in Supplementary Tables S2–S4 in the Supplementary Information.

## Results and Discussion

### Protein Design

**FIGURE 1:**
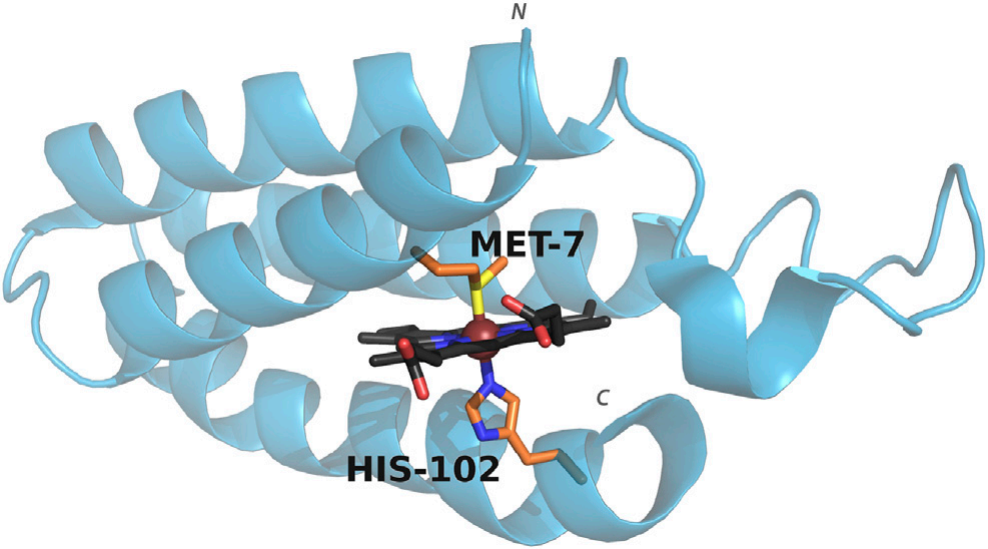
Structure of cyt *b*_562_ showing the coordinating axial ligands. PDB entry: 1QPU. Colors represent carbon (porphyrin: black, axial residues: orange), oxygen (red), nitrogen (blue), sulfur (yellow). The metal ion is shown as a sphere (brown). The *N* and *C* terminus are indicated.

Cyt *b*_562_ is a small, water-soluble, four-helix bundle that natively binds a heme cofactor via bis-axial ligation from the side chains of residues His102 and Met7 (Figure 1), and can coordinate a wide range of metalloporphyrins (Pia et al., 2012; Sommer et al., 2016; Bowen et al., 2020). Swapping in CoPPIX for heme and assessing activity under photocatalytic conditions results in a 10 fold increase in hydrogen production compared to CoPPIX in solution (Sommer et al., 2016). In order to facilitate binding of carbon dioxide and conversion to product, we utilized mutants that either remove axial ligation sites (M7A and H102A) or alter axial ligation (M7H) (Kamiya et al., 2001; Hay and Wydrzynski, 2005; Sommer et al., 2016). We assessed the effect of these mutations on the efficiency and specificity of CO_2_ reduction in water by cobalt porphyrins, which yields formate, carbon monoxide, and hydrogen in photocatalytic conditions.

We investigated the secondary structure and stability of the mutant proteins in the apo state and in response to binding to CoPPIX. The CD spectra of all apo mutants showed the typical signals of α-helical proteins, with local minima at 208 and 222 nm (Supplementary Information, Supplementary Figure S1). Upon reconstitution with CoPPIX, WT and M7A show minimal changes in this region, indicating that incorporation of the cofactor has little effect on the structure of the protein at room temperature. The signal for M7H increases upon addition of the porphyrin, showing that incorporation of the ligand stabilizes the secondary structure of the protein, possibly because coordination to the metal reduces the buried positive charges related to the two abutting histidines in the unoccupied active site. In contrast, H102A shows a decrease in signal upon addition of the porphyrin suggesting that this mutant adopts different apo and bound structures compared to the other mutants in the series (see discussion below).

More information on structural changes upon binding was gathered by comparing the stability of the mutants in the apo and holo state. We carried out unfolding experiments by monitoring the loss of intensity of the 222 nm peak in the CD spectrum at increasing temperature, obtaining thermal denaturation curves of the apo and holo mutants (SI, Supplementary Figure S1). The data presented in Table 1 shows that the apparent midpoint of the thermal denaturation, *T*_m_, increases significantly upon binding of the porphyrin, with the exception of H102A, which showed a minimal change in its *T*_m_ value.

**TABLE 1:** CD and binding data for Co-cyt *b*_562_ mutants. CD spectra was obtained at 20 °C in 10 mM phosphate buffer pH 7.5 with a protein concentration of 5 μM and CoPPIX concentration of 20 μM. The *K*_d_ values were obtained using UV-Vis spectroscopy by titrating a stock solution of apo protein to a solution of CoPPIX of known concentration (see Methods and Supplementary Figures S1 and S2).

Mutant	*T*_m_ (°C)		Δ*T*_m_ (°C)	*K*_d_ (nM)
	Apo	Holo		
WT	56	72	+16	45 ± 19
M7A	52	65	+13	175 ± 22
M7H	50	75	+25	559 ± 89
H102A	57	56	−1	81 ± 54

The stability of the apo state is strongly affected by mutations: compared to WT, the *T*_m_ of M7A is lowered 4 °C, possibly due to a decrease in core hydrophobic volume, balanced by the high helical propensity of alanine compared to methionine (Chakrabartty et al., 1991). M7H experiences a 6 °C destabilization. The solution structure of WT apo cyt *b*_562_ (Feng et al., 1994) shows that His7 is largely solvent accessible, suggesting a p*K*_a_ close to neutrality and indicating that the side chain can be partially charged under the experimental conditions (Edgcomb and Murphy, 2002). The observed destabilization correlates to lower helical propensity of histidine—particularly when ionized—compared to methionine (Pace and Scholtz, 1998). In contrast, H102A displays stability slightly higher than WT, because of the high helical propensity of alanine. Binding to CoPPIX stabilizes the proteins to an extent that reflects the changes in axial ligation: M7A is stabilized by 13 °C, a lesser extent than observed in WT (16 °C) because of the loss of axial ligand, while M7H experiences much higher stabilization (25 °C) because of the strong double axial ligation afforded by histidine, and the loss of buried charges upon coordination. In H102A, however, binding to CoPPIX does not increase stability. We speculate that comparable helical content and stabilities between the apo forms of H102A and WT arise from the small contribution of the C-terminal α-helix to the folding of apo cyt *b*_562_, because the C-terminus is unfolded in solution (PDB 1APC) (Feng et al., 1994). This observation suggests that folding of the C-terminal α-helix in the holo state of WT is predominantly driven by ligation of His102 to the porphyrin, and abolishing this interaction in H102A prevents folding of the bundle. In contrast, mutations to the axial position M7 have minimal effect on the stability of the holo protein: M7A is slightly destabilized compared to WT, while M7H displays higher apparent *T*_m_, reflecting the relative strength of the axial coordination.

All mutants bind CoPPIX with *K*_d_ values in the low to mid-nanomolar range, as assessed through UV-Vis titration of apo protein to a solution of CoPPIX (SI, Supplementary Figure S2 and Table 1). However, displacement of either one of the native axial ligands weakens affinity, and all mutants bind CoPPIX with higher *K*_d_ values than WT. Among the mutants, H102A displays the highest affinity, similar to WT, followed by M7A; M7H registers the weakest affinity by far. This observation suggests that binding of the cofactor requires a pre-structured binding pocket in the apo state, as the affinity trend mirrors the trend observed for the stability of the apo forms.

### Photocatalytic Activity

The Co-cyt *b*_562_ mutants were assayed to investigate their ability to reduce CO_2_ under photoinduced conditions by using [Ru (bpy)_3_]^2+^ as photosensitizer and ascorbic acid as sacrificial electron donor. The excited state of [Ru(bpy)_3_]^2+^ is reduced by ascorbic acid to [Ru(bpy)_3_]^+^, which in turns transfers electrons to the catalyst resulting in substrate reduction (Morris et al., 2009). The Co-cyt *b*_562_ mutants produced carbon monoxide and formate, in addition to H_2_ produced both in the presence and absence of CO_2_. Control experiments lacking CoPPIX showed little H_2_ was produced and no CO or HCO_2_^−^ were detected, indicating that these species were produced by the cobalt catalyst. The reactivity observed is in line with previous mechanistic studies of cobalt porphyrins used as catalysts for these reactions (SI, Supplementary Figure S3) (Dhanasekaran et al., 1999; Morris et al., 2009; Leung et al., 2010; Nielsen and Leung, 2010; Shen et al., 2016; Call et al., 2019b).

**FIGURE 2:**
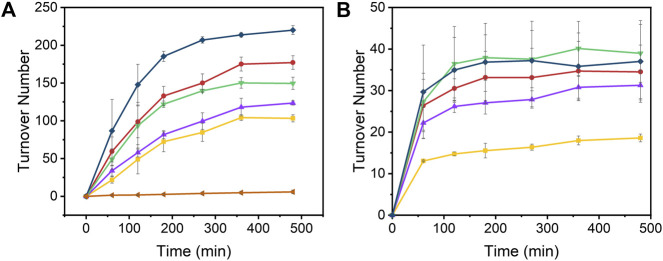
Turnover number values of **(A)** H_2_ and **(B)** CO over time from the photoinduced reduction of protons by [Ru(bpy)_3_]^2+^, CoPPIX, and Co-cyt *b*_562_ mutants at pH 6.0 under 1 atm CO_2_. The experiments were carried out in 100 mM ascorbic acid, 1 mM [Ru(bpy)_3_]^2+^, 200 mM potassium phosphate, 20 µM CoPPIX (except in the negative control), and 30 µM of corresponding apo cyt *b*_562_ (except for free CoPPIX; apo WT cyt *b*_562_ was used for the negative control) under white LED light irradiation. Colors represent [Ru(bpy)_3_]^2+^ (negative control, orange side triangles), free CoPPIX (yellow squares), WT (red circles), M7A (purple up triangles), M7H (green down triangles), and H102A (dark blue diamonds). The TON for the negative control was calculated assuming a hypothetical catalyst content identical to the other samples (8 nmol) for direct comparison. The experiments were run on triplicate and error bars represent the standard deviation of the sample.

**FIGURE 3:**
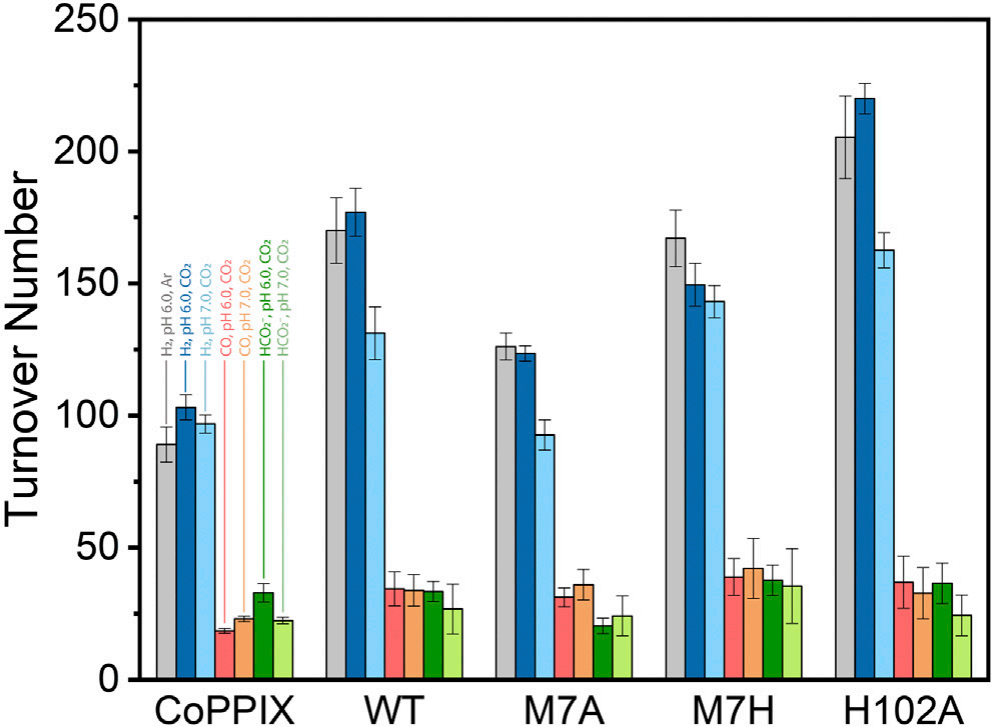
Turnover number values obtained for CoPPIX and cobalt cyt *b*_562_ mutants under irradiation with light for 8 h in 100 mM ascorbic acid, 1 mM [Ru(bpy)_3_]^2+^, and 200 mM potassium phosphate. The bars represent the TON of each catalyst at pH 6.0 in absence (H_2_: gray) and presence of CO_2_ (H_2_: dark blue, CO: red, HCO_2_^−^: dark green); and in the presence of CO_2_ at pH 7 (H_2_: light blue, CO: orange, HCO_2_^−^: light green). The experiments were run on triplicate and error bars represent the standard deviation of the sample.

The time-dependent results of the experiments are presented in Figure 2 and the final turnover numbers are shown on Table 2 and Figure 3. When assayed under a CO_2_ atmosphere at pH 6.0, all mutants showed a two-fold increase in production of CO equivalent compared to the porphyrin alone, indicating that activity is increased by the interaction of the protein with the porphyrin (Table 2; Figures 2, 3). In contrast, activity toward formate production was similar to free porphyrin in all cases, with a slight decrease for M7A at pH 6.0. Observed H_2_ production activity at pH 6.0 was in all cases higher than CoPPIX, however with significant variation between mutants: M7A showed a (20 ± 5)% increase in H_2_ compared to the porphyrin alone, followed by M7H ((45 ± 7)%) and WT ((72 ± 7)%, *p* = 0.64 for the M7H and WT comparison), and finally H102A with the highest increase in activity [(113 ± 5)%]. The assay was repeated in the absence of CO_2_ (Figure 3 and SI, Supplementary Figure S6), and yielded similar TON values for H_2_, indicating that overall H_2_ production is not affected by the presence of CO_2_. Methionine coordination at position seven results in a higher overall activity, as mutants bearing this residue (WT, H102A) resulted in the highest TON values. A possible explanation of these observations is that as the metal center becomes more electron rich, a softer ligand—such as methionine’s thioether side chain—is capable of better stabilizing the reduced intermediates that are formed throughout the catalytic cycle. Finally, the difference between the activities of H102A and WT could be due to the opening of a coordination site on one side of the porphyrin, thus increasing the chance of substrate binding to the active site. The near identical TONs observed for CO production as opposed to the variability observed for proton reduction suggests that the protein scaffold may provide proton relay pathways toward the active site. The rate limiting step of CO_2_ reduction to CO does not involve protonation steps, thus not benefiting in the same manner from the different proton environments as the H_2_-production cycle (Supplementary Figure S3).

**TABLE 2:** Turnover numbers for the observed products under light irradiation in 200 mM potassium phosphate, 100 mM ascorbic acid, 1 mM [Ru(bpy)_3_]^2+^, and 1 atm of gas.

Mutant	TON H_2_			TON CO		TON HCO_2_^–^	
	pH 6^a^	pH 6	pH 7	pH 6	pH 7	pH 6	pH 7
CoPPIX	89 ± 6	103 ± 5	97 ± 3	19 ± 1	23 ± 1	33 ± 4	22 ± 1
WT	170 ± 12	177 ± 9	131 ± 10	35 ± 6	34 ± 6	33 ± 4	27 ± 9
M7A	126 ± 5	124 ± 3	93 ± 6	31 ± 4	36 ± 6	20 ± 3	24 ± 8
M7H	167 ± 10	150 ± 8	143 ± 6	39 ± 7	42 ± 11	38 ± 6	35 ± 14
H102A	205 ± 16	220 ± 6	163 ± 7	37 ± 10	33 ± 10	37 ± 8	25 ± 8

^a^ Control experiment under Ar instead of CO_2_.

The turnover numbers relative to CO production measured at pH 7.0 were generally unchanged for the holoprotein, while a slight increase was recorded for CoPPIX. Unsurprisingly the H_2_ yield for WT, M7A, and H102A decreased by 20%, as proton concentration is decreased 10-fold. However, no statistically significant change was observed for the H_2_ TON of the free porphyrin or the M7H mutant. Finally, a decrease in formate production was observed for the free porphyrin, consistent with the fact that its formation is dependent on proton concentration. The formate TON values for the holo proteins were not statistically different to those obtained at pH 6.0, suggesting that the protein environment might serve as a proton source throughout this particular catalytic route, thus mitigating the effect of the lower proton concentration in solution.

## Conclusion

We have investigated the effect of the primary coordination sphere provided by the protein environment of cobalt cytochrome *b*_562_ in the light-promoted CO_2_ reduction pathway in mild aqueous conditions. Our results indicate that the native CO_2_ reduction activity of CoPPIX is increased when incorporated into the protein, whereas HCO_2_^−^ production is not affected by the protein scaffold. Additionally, proton reduction showed a difference in activities between the mutants, suggesting that both electronic and steric effects play an important role throughout the catalytic cycle that leads to H_2_ production. Three of our protein scaffolds (WT, M7A, H102A) displayed pH dependance of proton reduction activity as expected, whereas M7H and CoPPIX did not show any noticeable change in activity at the examined pH values. No pH dependence of CO_2_ reduction activity was observed between the catalysts, indicating that other factors may come into play in the mechanism of CO_2_ reduction by these catalysts.

This work illustrates the first example of light driven carbon dioxide reduction by a protein-cobalt porphyrin catalyst, and demonstrates that hybrid metalloenzymes can be generated by incorporating CoPPIX into cyt *b*_562_; in water, the novel enzyme yields CO, formate, and hydrogen. Hydrogen production activity is modulated by mutating residues involved in the first coordination sphere of the porphyrin; in contrast, the mutations investigated here do not affect significantly CO_2_ reduction. Further work explanding mutations in the first and second coordination sphere could provide insight into the mechanism(s) of photoinduced reduction of H^+^ and CO_2_, which in turn could result in better catalysts for renewable energy and green chemistry.

## Data Availability

The original contributions presented in the study are included in the article/Supplementary Material, further inquiries can be directed to the corresponding author.
