# Supplementary material for: Light-Driven CO2 Reduction by Co-Cytochrome b 562
Source: Front Mol Biosci. 2021 Apr 15;8:609654. doi: 10.3389/fmolb.2021.609654 (PMC8082397; doi:10.3389/fmolb.2021.609654)
Supplement: Supplementary file 1 [file datasheet1.pdf]

Supplementary Information

Light-driven CO_2 reduction by Co-Cytochrome b_{562}

Rafael Alcala-Torano[1], Nicholas Halloran[1], Noah Gwerder[1], Dayn J. Sommer[1], Giovanna Ghirlanda[1*]

[1]School of Molecular Sciences, Arizona State University, Tempe, AZ,

*** Correspondence:**
Giovanna Ghirlanda
Giovanna.Ghirlanda@asu.edu

1. Derivation of fitting equation for the titration data

Since the Soret peaks of CoPPIX and holo protein overlap with each other, the absorbance A at the Soret wavelength of the holo construct (~425 nm) can be expressed as:

$$A = \varepsilon_{CoPPIX}[CoPPIX] + \varepsilon_{CoCyt}[CoCyt] \tag{S1}$$

where ε_i is the extinction coefficient of species *i* at this wavelength, [X] is the molar concentration of species X, and CoCyt indicates holo protein. When no protein has been added, equation S1 becomes:

$$A_0 = \varepsilon_{CoPPIX}[CoPPIX]_T \tag{S2}$$

where A_0 indicates the initial absorbance of free CoPPIX at the Soret band of the holo protein (~425 nm), and $[CoPPIX]_T$ corresponds to the total CoPPIX concentration. Subtracting equation S2 from equation S1 and simplifying we obtain:

$$A - A_0 = \varepsilon_{CoPPIX}([CoPPIX] - [CoPPIX]_T) + \varepsilon_{\mathrm{CoCyt}}[CoCyt] \tag{S3}$$

Further, for the dissociation equilibrium of CoCyt shown below, we can write the mass balance equation for CoPPIX as equation S4:

$$CoCyt \rightleftharpoons CoPPIX\ +\ Cyt$$

$$[CoPPIX]_T = [CoPPIX] + [CoCyt] \tag{S4}$$

By substituting the value of $[CoPPIX]_T$ from equation S4 into equation S3, we can simplify as:

$$\Delta A = \Delta\varepsilon[CoCyt] \tag{S5}$$

Where $\Delta A = A - A_0$ and $\Delta\varepsilon = \varepsilon_{CoCyt} - \varepsilon_{CoPPIX}$. The K_d expression for the dissociation of CoCyt is given by equation S6, and the mass balance equation for protein is shown in equation S7:

$$K_d = \frac{[\mathrm{CoPPIX}][\mathrm{Cyt}]}{[\mathrm{CoCyt}]} \tag{S6}$$

$$[\mathrm{Cyt}]_\mathrm{T} = [\mathrm{Cyt}] + [\mathrm{CoCyt}] \tag{S7}$$

Making use of mass balance equations S4 and S7, equation S6 becomes:

$$K_d = \frac{([CoPPIX]_T - [CoCyt])([Cyt]_T - [CoCyt])}{[CoCyt]} \tag{S8}$$

Simplifying this equation and factoring [CoCyt], we get:

$$[CoCyt]^2 - ([Cyt]_T + [CoPPIX]_T + K_d)[CoCyt] + [CoPPIX]_T[Cyt]_T = 0 \tag{S9}$$

Using the quadratic formula, we can solve for [CoCyt] as:

$$[CoCyt] = \frac{1}{2}\Big([Cyt]_T + [CoPPIX]_T + K_d \pm \sqrt{([Cyt]_T + [CoPPIX]_T + K_d)^2 - 4[CoPPIX]_T[Cyt]_T}\Big) \tag{S10}$$

Given that [CoCyt] cannot be larger than $[CoPPIX]_T$, the only valid solution is that with the negative radical:

$$[CoCyt] = \frac{1}{2}\Big([Cyt]_T + [CoPPIX]_T + K_d - \sqrt{([Cyt]_T + [CoPPIX]_T + K_d)^2 - 4[CoPPIX]_T[Cyt]_T}\Big) \qquad \text{(S10)}$$

Finally, substituting the value of [CoCyt] from equation S5 and solving for ΔA we obtain the desired equation:

$$\Delta A = \frac{\Delta\varepsilon}{2}\Big([Cyt]_T + [CoPPIX]_T + K_d - \sqrt{([Cyt]_T + [CoPPIX]_T + K_d)^2 - 4[CoPPIX]_T[Cyt]_T}\Big) \qquad \text{(1)}$$

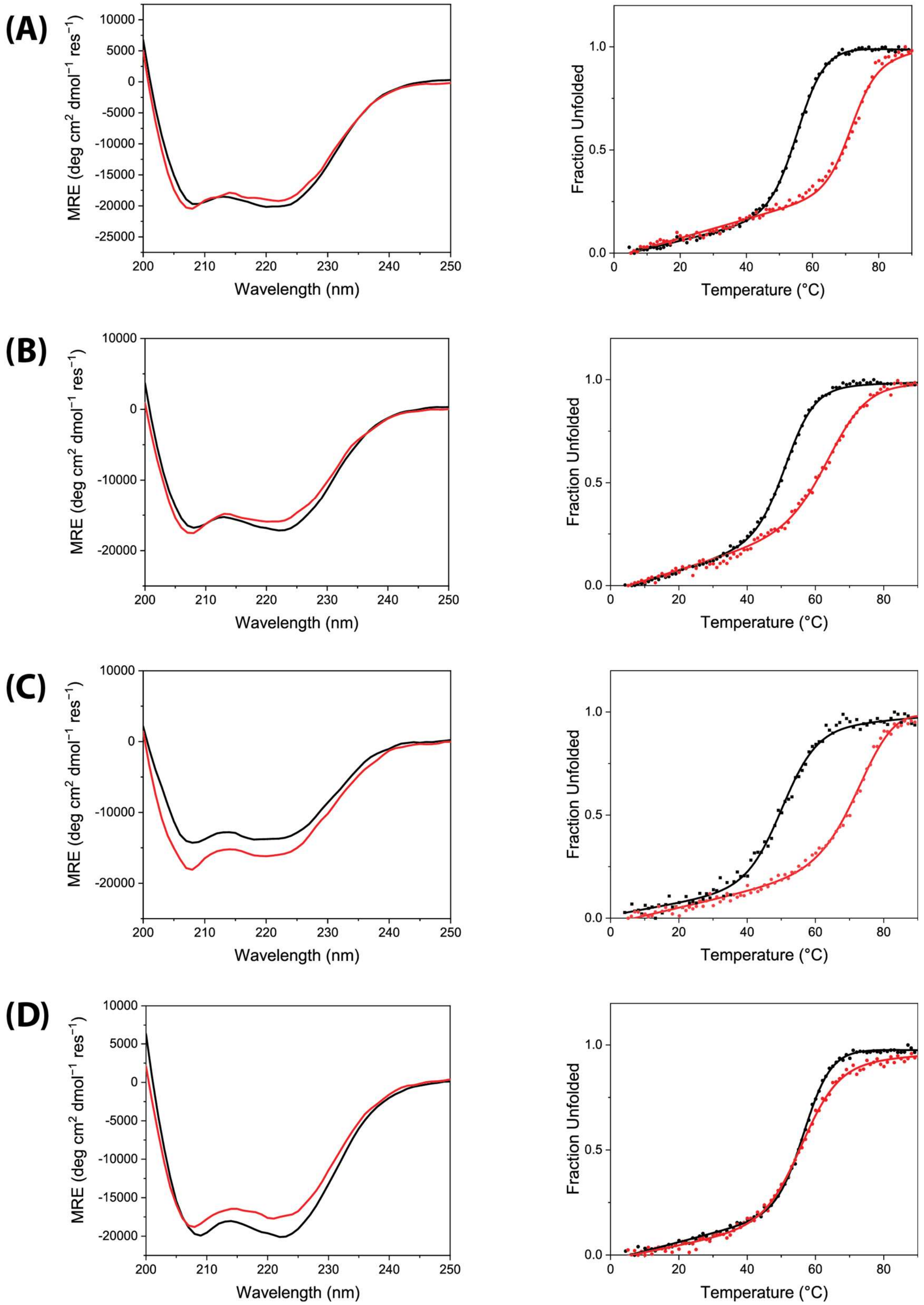

Figure S1. CD spectra (left) and thermal denaturation curves (right) of apo (black) and holo (red) cobalt cytochrome b_{562} (A) WT, (B) M7A, (C) M7H, and (D) H102A.

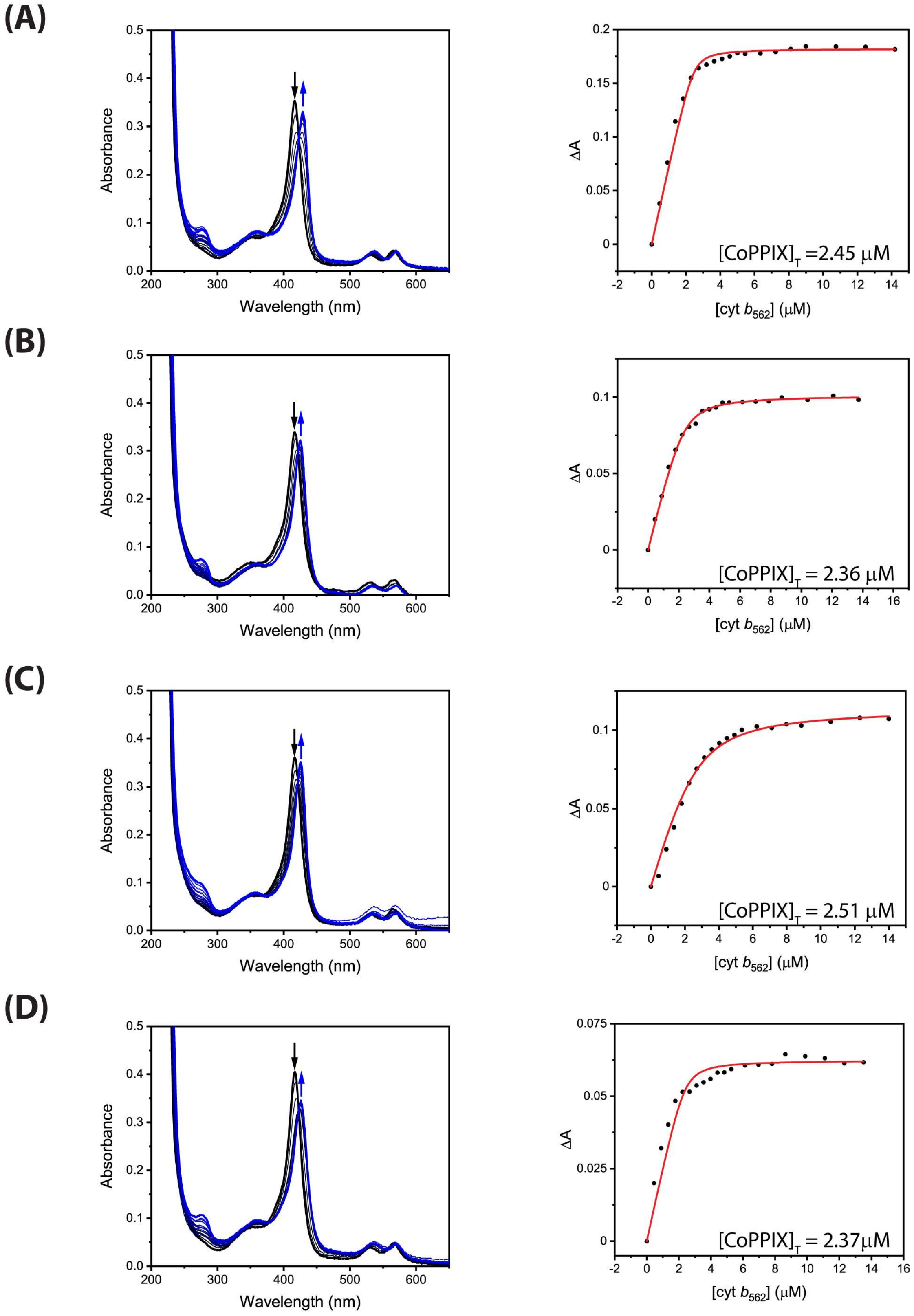

Figure S2. UV-Vis traces (left) and fitted binding isotherms (right) for the titration of free CoPPIX (initial black trace) with cyt b_{562} (A) WT, (B) M7A, (C) M7H, and (D) H102A in 200 mM potassium phosphate pH 7.5. The blue trace indicates the endpoint of the titration. The arrows indicate the changes occurring upon addition of titrant.

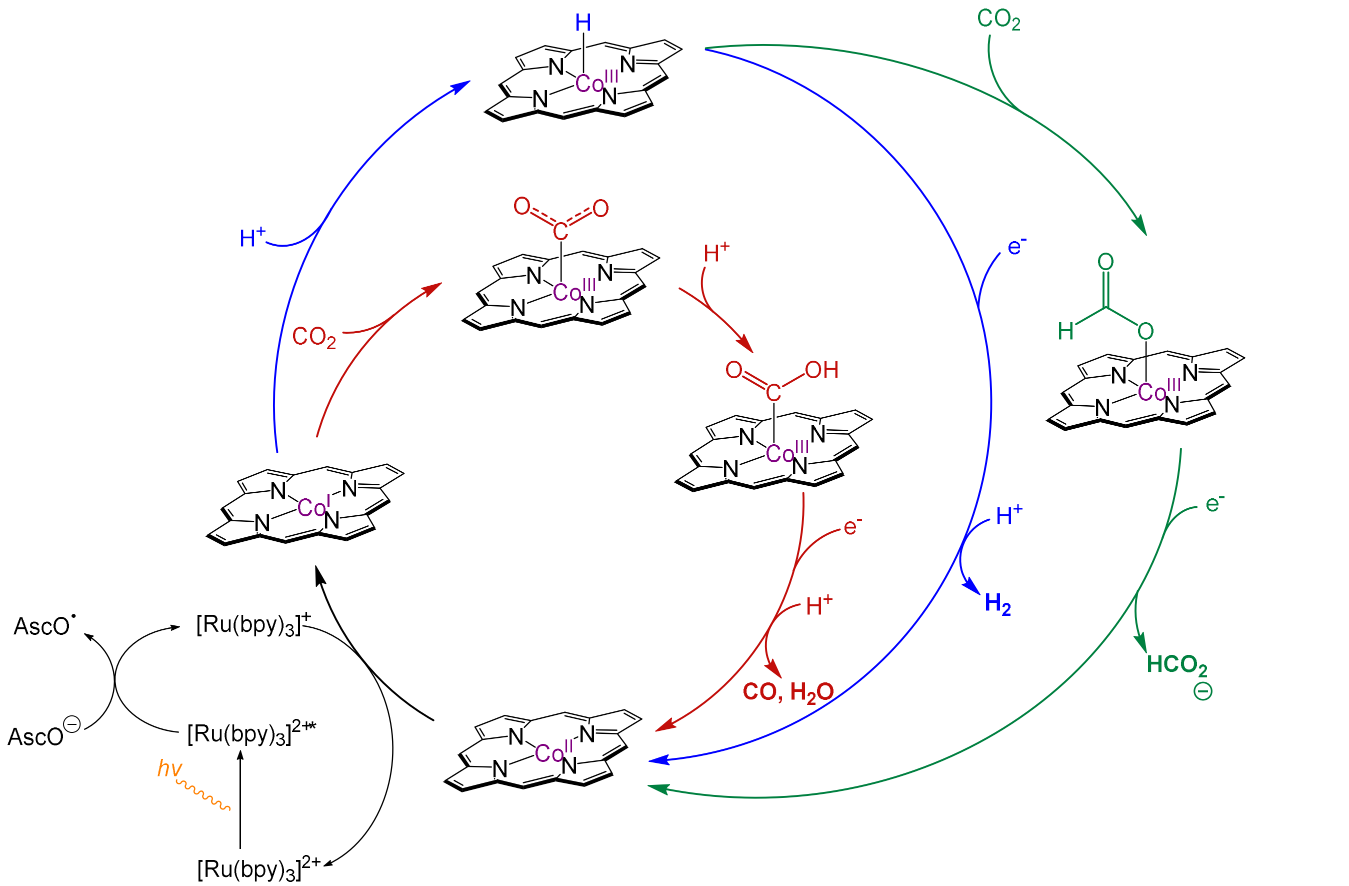

Figure S3. Catalytic cycles for the generation of H_2 (blue), CO (red), and HCO_2^- (green) by cobalt porphyrins through photoirradiation in the presence of $[Ru(bpy)^3]^{2+}$ and ascorbate (Asc^-) as sacrificial electron donor.

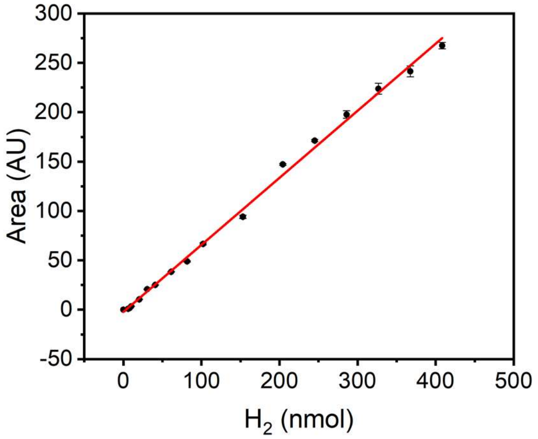

Figure S4. Calibration curve for H_2. The data was fitted to the linear equation y = 0.679x – 2.27, with an adjusted $R^2 = 0.9970$.

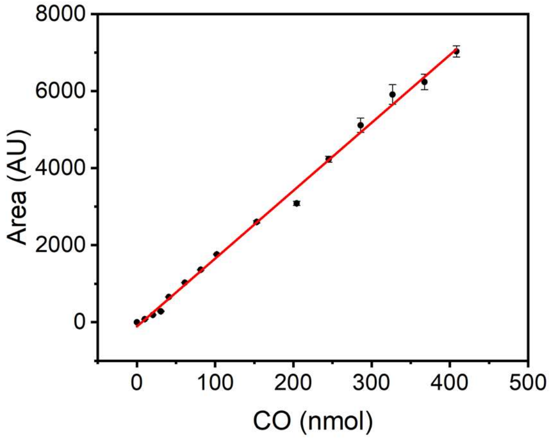

Figure S5. Calibration curve for CO. The data was fitted to the linear equation y = 17.62x − 107.97, with an adjusted $R^2 = 0.9958$.

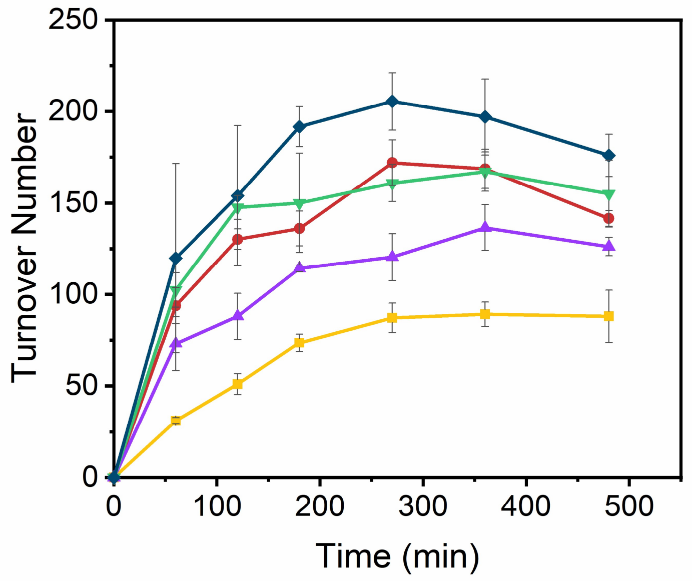

Figure S6. Produced H_2 and over time from the photoinduced reduction of protons by CoPPIX and Co-cyt *b*$_{562}$ mutants at pH 6.0 under argon. The experiments were carried out in 100 mM ascorbic acid, 1 mM $[Ru(bpy)_3]^{2+}$, 200 mM potassium phosphate, and 20 μM catalyst under white LED light irradiation. Colors represent free CoPPIX (yellow squares), WT (red circles), M7A (purple up triangles), M7H (green down triangles), and H102A (dark blue diamonds). The experiments were carried out in triplicate. The error bars represent the standard deviation of the sample.

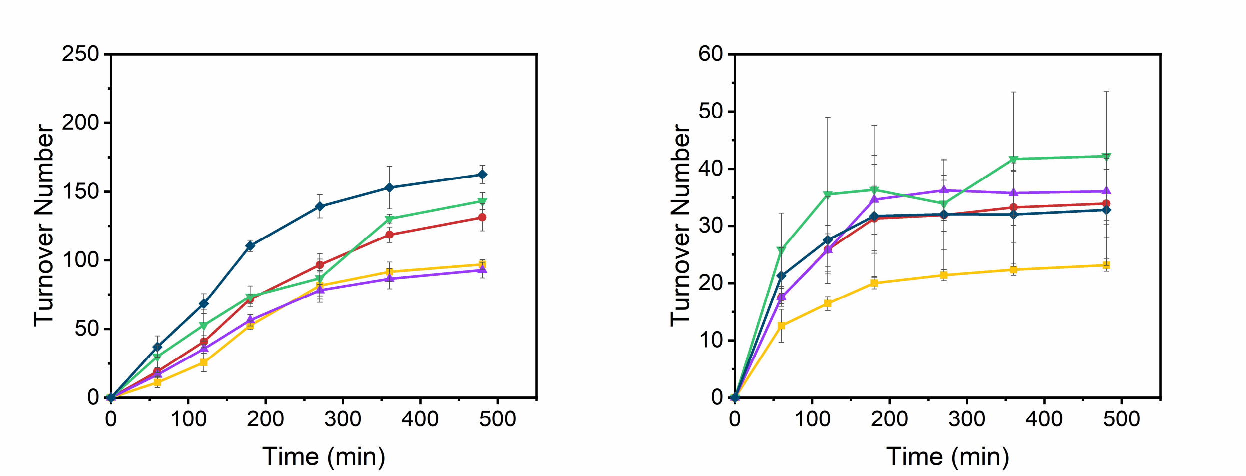

Figure S7. Produced H_2 (left) and CO (right) and over time from the photoinduced reduction of protons by CoPPIX and Co-cyt b_{562} mutants at pH 7.0 under 1 atm CO_2. The experiments were carried out in 100 mM ascorbic acid, 1 mM $[Ru(bpy)_3]^{2+}$, 200 mM potassium phosphate, 20 µM CoPPIX, and 30 µM apo cyt b_{562} (except for CoPPIX) under white LED light irradiation. Colors represent free CoPPIX (yellow squares), WT (red circles), M7A (purple up triangles), M7H (green down triangles), and H102A (dark blue diamonds). Experiments were carried out in triplicate. The error bars represent the standard deviation of the sample.

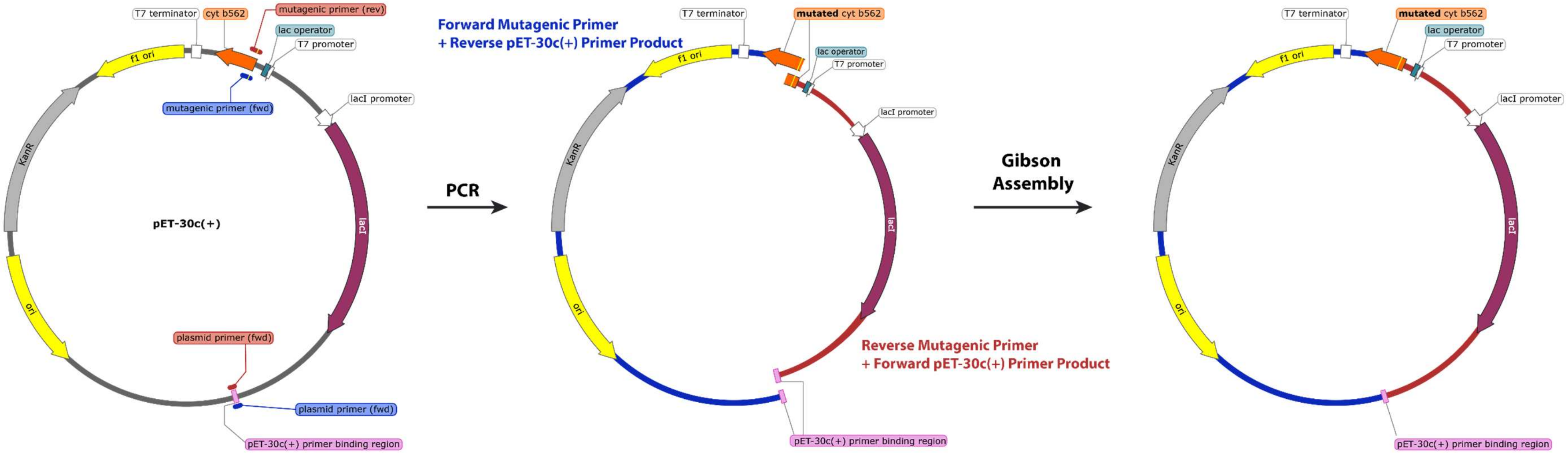

Figure S8. Scheme illustrating the generation of mutants in a a pET30c(+) vector encoding WT cyt b_{562} via Gibson Assembly. The region encoding for cyt b_{562} is shown as an orange feature on the plasmid map, and an auxiliary plasmid primer binding region is shown in purple. Primers are depicted as blue or red lines offset from the plasmid map. A yellow line indicates the desired mutation. For each mutant, two PCR reactions were carried out. The first product consists in the PCR reaction using the forward mutagenic primer and the reverse pET-30c(+) primer (blue product), and the second PCR product using the opposite combination (red product). The PCR products were purified by gel electrophoresis, and the circular vector was then obtained by mixing both fragments in presence of the Gibson Assembly Master Mix (NEB) according to the manufacturer's recommendations.

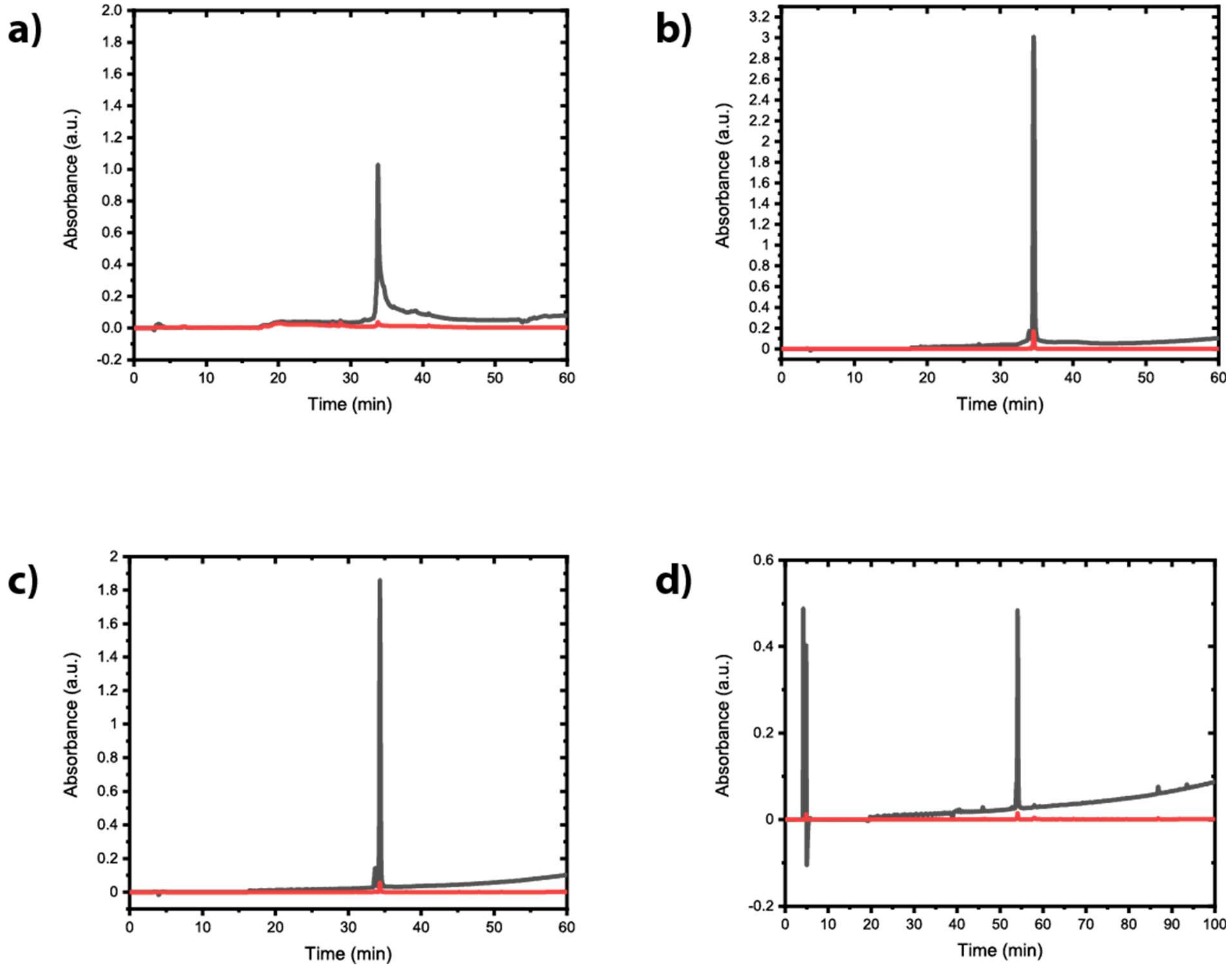

Figure S9. Analytical HPLC traces of apo cytochrome b_{562} constructs monitored by UV-Vis at 220 nm (black traces) and 280 nm (red traces). a) WT, b) M7A, c) M7H, d) H102A.

Table S1. Primers used for generation of mutants by PCR. All primers are shown in a 5′ to 3′ direction (left to right).

Mutant	Forward	Reverse
pET30c(+)	CGATGCAGATCCGGAACATAATG	CATTATGTTCCGGATCTGCATCG
M7A	GAAGACAACGCGGAAACCCTG	CAGGGTTTCCGCGTTGTCTTC
M7H	GAAGACAACCACGAAACCCTG	CAGGGTTTCGTGGTTGTCTTC
H102A	GAAATGCTTACGCGCAGAAGTATC	GATACTTCTGCGCGTAAGCATTTC

Table S2. Calculated *p*-values for each comparison between mutants at each examined condition. The null hypothesis for each test was H_0: $\bar{x}_1 - \bar{x}_2 = 0$. Values corresponding to $p > 0.05$ (the null hypothesis holds) are highlighted.

	**CoPPIX**	**WT**	**M7A**	**M7H**	**H102A**
**pH 6.0 under Ar**					
Hydrogen gas					
CoPPIX		5.2×10^{-4}	4.5×10^{-3}	4.3×10^{-5}	2.9×10^{-4}
WT			0.025	0.64	0.044
M7A				0.032	4.0×10^{-3}
M7H					0.025
**pH 6.0 under CO_2**					
Hydrogen gas					
$[Ru(bpy)_3]^{2+}$	1.9×10^{-5}	9.7×10^{-6}	4.1×10^{-6}	1.4×10^{-5}	1.1×10^{-6}
CoPPIX		2.4×10^{-4}	3.2×10^{-3}	1.0×10^{-3}	1.1×10^{-5}
WT			6.2×10^{-4}	0.017	2.3×10^{-3}
M7A				6.4×10^{-3}	1.4×10^{-5}
M7H					2.6×10^{-4}
Carbon monoxide					
CoPPIX		0.048	3.9×10^{-3}	0.035	0.082
WT			0.49	0.46	0.73
M7A				0.16	0.40
M7H					0.79
Formate					
CoPPIX		0.88	9.8×10^{-3}	0.30	0.52
WT			8.9×10^{-3}	0.34	0.57
M7A				9.5×10^{-3}	0.028
M7H					0.83
**pH 7.0 under CO_2**					
Hydrogen gas					
CoPPIX		2.4×10^{-4}	3.2×10^{-3}	1.0×10^{-3}	1.1×10^{-5}
WT			6.2×10^{-4}	0.017	6.1×10^{-3}
M7A				6.4×10^{-3}	1.4×10^{-5}
M7H					2.6×10^{-4}
Carbon monoxide					
CoPPIX		0.037	0.019	0.099	0.23
WT			0.68	0.33	0.87

	CoPPIX	WT	M7A	M7H	H102A
M7A				0.45	0.64
M7H					0.34
Formate					
CoPPIX		0.25	0.50	0.084	0.46
WT			0.50	0.15	0.54
M7A				0.071	0.94
M7H					0.076

Table S3. Calculated *p*-values for each comparison between conditions for each evaluated mutant. Condition A: pH 6.0 under Ar; condition B: pH 6.0 under CO_2; condition C: pH 7.0 under CO_2. The null hypothesis for each test was H_0: $\bar{x}_1 - \bar{x}_2 = 0$. Values corresponding to $p > 0.05$ (the null hypothesis holds) are highlighted.

Mutant	Hydrogen gas			Carbon monoxide	Formate
	**A vs B**	**A vs C**	**B vs C**	**B vs C**	**B vs C**
CoPPIX	0.040	0.43	0.14	5.3×10^{-3}	7.0×10^{-3}
WT	0.60	0.011	4.1×10^{-3}	0.91	0.78
M7A	0.16	5.4×10^{-3}	1.1×10^{-3}	0.29	0.24
M7H	0.086	0.028	0.34	0.69	0.80
H102A	0.20	0.012	3.6×10^{-4}	0.63	0.072

Table S4. Calculated p-values for the comparison between the estimated K_d values (in nM). The null hypothesis for each test was H_0: $K_{d,1} - K_{d,2} = 0$. Values corresponding to $p > 0.05$ (the null hypothesis holds) are highlighted.

	**M7A**	**M7H**	**H102A**
**WT**	9.0×10^{-22}	2.3×10^{-25}	7.7×10^{-3}
**M7A**		8.7×10^{-21}	1.3×10^{-8}
**M7H**			3.6×10^{-22}
